# The causal relationship between systemic lupus erythematosus and juvenile myoclonic epilepsy: A Mendelian randomization study and mediation analysis

**DOI:** 10.1002/ibra.12191

**Published:** 2025-01-07

**Authors:** Sirui Chen, Ningning Zhang, Ruirui Zhang, Lan Zhang, Dadong Luo, Junqiang Li, Yaqing Liu, Yunan Wang, Xinyue Duan, Xin Tian, Tiancheng Wang

**Affiliations:** ^1^ The Second Hospital & Clinical Medical School Lanzhou University Lanzhou China; ^2^ Department of Neurology, Epilepsy Center, The Second Hospital & Clinical Medical School Lanzhou University Lanzhou China; ^3^ The First Clinical Medical College Chongqing Medical University Chongqing China; ^4^ Department of Neurology The First Affiliated Hospital of Chongqing Medical University, Chongqing Key Laboratory of Neurology Chongqing China; ^5^ Key Laboratory of Major Brain Disease and Aging Research (Ministry of Education) Chongqing Medical University Chongqing China

**Keywords:** causality, inflammatory cytokines, juvenile myoclonic epilepsy, Mendelian randomization, systemic lupus erythematosus

## Abstract

This study aimed to investigate the causal relationship between systemic lupus erythematosus (SLE) and juvenile myoclonic epilepsy (JME). Univariable and reverse Mendelian randomization (MR) analyses were performed to investigate the potential causal associations between SLE, systemic autoimmune disorders (SADs), and JME. Two‐step mediation MR analysis was further performed to explore indirect factors that may influence the relationship between SLE and JME. Summary data on SADs were extracted from the Integrative Epidemiology Unit Open genome‐wide association study database, and summary statistics for JME were acquired from the International League Against Epilepsy Consortium. The inverse‐variance weighted (IVW) method was used for primary analysis, supplemented by MR‐Egger and weighted median. In the univariable MR analysis, IVW results indicated a causal relationship between SLE and an increased risk of JME (odds ratio = 1.0030, 95% confidence interval, 1.0004–1.0057; *p* = 0.023). The subsequent mediation MR analysis showed that inflammatory cytokines may not be the mediating factors between SLE and JME, while the inverse MR analysis found no significant relationship. Our study indicated that genetic susceptibility to SLE was causally linked to JME. However, subsequent mediation analysis failed to identify the potential mediators that could influence this relationship. Moreover, evidence suggested that other SADs were not causally associated with JME. This study may provide guidance for screening risk factors for seizures and exploring potential treatments in SLE and JME, and even all SADs and JME.

## Graphical abstract

Mendelian randomization (MR) studies were conducted using the inverse‐variance weighted (IVW) method, MR‐Egger and weighted median on juvenile myoclonic epilepsy (JME), and systemic lupus erythematosus (SLE) data from the Integrative Epidemiology Unit (IEU) Open genome‐wide association study (GWAS) database and the International League Against Epilepsy (ILAE) Consortium. Research suggested a causal relationship between the genetic susceptibility of SLE and JME, and there was almost no evidence in reverse MR analysis to suggest a causal genetic relationship between JME and SLE. There may be a potential connection between SLE and JME, in which antineuronal antibodies, inflammatory cytokines, or genetic factors may play an important role. There was no relationship between other systemic autoimmune diseases and JME. Other evidence suggested that inflammatory cytokines were not causally linked to JME. This study may provide guidance for screening risk factors for seizures and exploring potential treatments in SLE and JME, and even all systemic autoimmune disorders and JME.

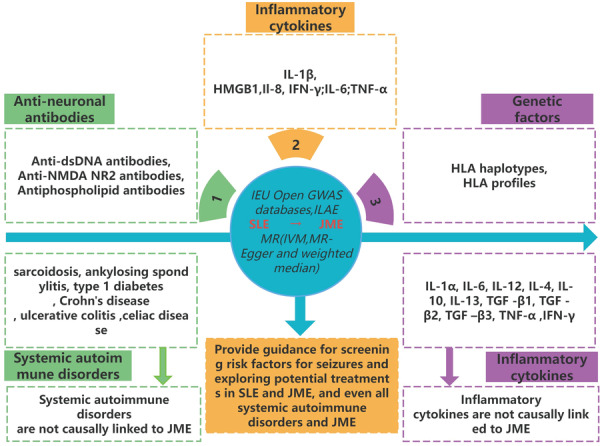

## 1 INTRODUCTION

The relationship between systemic autoimmune disorders (SADs) and epilepsy has long been the focus of attention. A meta‐analysis investigating the relationship between SADs and epilepsy found that patients with SADs had a 2.5 times higher risk of epilepsy, while patients with epilepsy had a 2.5 times higher risk of SADs.^1^ The correlation between SADs and epilepsy was also found to be stronger in the population aged <20 years, indicating the possibility of a common pathogenesis.^1^, ^2^ Multiple SADs are associated with an increased risk of epilepsy, including systemic lupus erythematosus (SLE), sarcoidosis, ankylosing spondylitis, type 1 diabetes mellitus (T1DM), Crohn's disease (CD), ulcerative colitis (UC), and celiac disease. However, the clear relationship between SADs and epilepsy has not yet reached a consensus.^2^, ^3^, ^4^ This may be due to the heterogeneous association between epilepsy and certain SADs or from a lack of research on this association.

SLE is one of the common SADs.^5^ SLE affects the central nervous system through direct neuronal damage, cerebrovascular damage, or pathogenic mechanisms.^2^, ^6^ The most common type of seizure in SLE is the generalized tonic‐clonic seizure, and the electroencephalogram results are usually observed as nonspecific with background slowing. The overall prognosis of SLE is good, with favorable response to antiepileptic drugs and immunosuppressive therapy.^7^ There is growing evidence of a strong relationship between SLE and epilepsy. Epileptic seizures are the most relevant clinical manifestation of SLE, usually occurring before the onset of the disease, and are associated with poor prognosis.^8^ Previous studies have shown that approximately 8% of patients with SLE experience seizures, and epilepsy and SLE may occur together due to genetic predisposition, including juvenile myoclonic epilepsy (JME).^9^, ^10^ JME is one of the most common childhood and adolescent epilepsy syndromes, accounting for approximately 5% of all epilepsy cases.^11^ JME is characterized by the presence of absence, myoclonus and generalized tonic‐clonic seizures, particularly in females.^11^, ^12^ Similar to JME, SLE is more common in young women, may cause seizures, and responds well to antiepileptic drugs. However, whether JME drives SLE or vice versa remains controversial.

Currently, there is little research on the correlation between SLE and JME; therefore, it is necessary to verify the true causal relationship. Contrarily, there may also be a common mechanism underlying these two diseases, in which autoantibodies and inflammatory cytokines may play a role.^5^ Further research on whether an association exists between SLE and JME may have important therapeutic implications for all SADs and ultimately enable the study of targeted therapy for patients with JME co‐suffering from SADs.

Mendelian randomization (MR) is a method that uses single nucleotide polymorphisms (SNPs) associated with exposure, to explore the causal effects of exposure factors on outcomes.^13^ This method not only limits the reverse causal relationship but also significantly reduces the possibility of residual confounding factors affecting the results.^14^ To this end, a bidirectional two‐sample MR study was performed to explore the causal relationships between SLE, other autoimmune diseases, and JME. Considering that inflammatory cytokines are common risk factors for both SLE and JME, we performed a mediation analysis to investigate whether SLE promotes JME by influencing inflammatory cytokines.

## 2 MATERIALS AND METHODS

### 2.1 Study design

**Figure 1:**
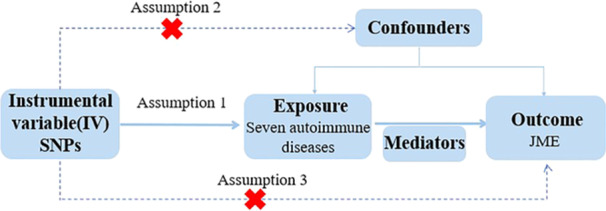
Overview and assumptions of the MR study design. Three major assumptions of the MR analysis are as follows: Assumption 1: Genetic variation as an instrumental variable must be genuinely associated with exposure (autoimmune diseases). Assumption 2: Exposure‐outcome confounders have no effect on genetic variation. Assumption 3: Genetic variation affects JME through autoimmune diseases only, independent of other pathways. JME, juvenile myoclonic epilepsy; MR, Mendelian randomization; SNPs, single nucleotide polymorphisms. [Color figure can be viewed at wileyonlinelibrary.com]

We conducted univariable and reverse MR analyses to investigate potential causal associations between SLE, SADs, and JME. Additionally, we employed a two‐step mediation MR analysis to further explore indirect factors that may influence the relationship between SLE and JME. The data used in this MR analysis were obtained from publicly available summarized genome‐wide association study (GWAS) data of European ancestry. MR must satisfy three fundamental assumptions (Figure 1): (1) genetic instruments are genuinely associated with exposure; (2) genetic instruments are not associated with confounders; and (3) genetic instruments influence the outcome only through exposure. A detailed flowchart of the study design is presented in Figure 1.

### 2.2 Data source

Summary data on SLE were extracted from the Integrative Epidemiology Unit (IEU) Open GWAS database (https://gwas.mrcieu.ac.uk/), including 5201 cases and 9066 controls.^15^ The data of other autoimmune diseases considered in our analysis included sarcoidosis, ankylosing spondylitis, T1DM, CD, UC, and celiac disease, which were also acquired from the IEU Open GWAS database.^16^, ^17^, ^18^ For inflammatory cytokines, data were obtained from the study by Suhre et al.,^19^ which included 11 cytokines. Summary statistics for JME were obtained from the International League Against Epilepsy Consortium (https://www.ilae.org/), which included 1181 cases and 29,677 controls with all participants of European ancestry.^20^ The detailed characteristics are shown in Supporting Information S1: Tables S1 and S2.

### 2.3 Selection of instrumental variables

First, we used a *p*‐value threshold of 5 × 10^–8^ to obtain SNPs significantly associated with SLE. We then removed linkage disequilibrium (*r* ^2^ < 0.001 and 1 Mb distance) to obtain independent SNPs. Meanwhile, F‐statistic (*β* ^2^/se^2^) was used to evaluate the strength of MR, and SNPs with an F‐statistic higher than 10 were included (Supporting Information S1: Tables S3 and S4). After excluding palindromic SNPs, the final screened SNPs were considered for further analysis. Additionally, SNPs with potential confounders (smoking, obesity, alcohol consumption, and diabetes) were excluded from the online PhenoScanner database (http://www.phenoscanner.medschl.cam.ac.uk/).

### 2.4 Statistical analysis

Inverse‐variance weighted (IVW) is a widely used method for MR estimation, which implies that all SNPs included in the analysis are valid, but also prone to pleiotropic bias. Other MR methods, such as MR‐Egger, weighted median, simple mode, and weighted mode, are applied as complementary methods. We used the odds ratio (OR) and 95% confidence interval (CI) to express the causal effects between SADs, inflammatory cytokines, and JME. All the above analyses were performed using the two‐sample MR (0.5.7) and MR‐PRESSO (1.0) packages in R version 4.3.0. A *p*‐value < 0.05 was considered evidence of statistically significant causality. We performed a series of sensitivity analyses, including heterogeneity and pleiotropy, to evaluate any bias in MR assumptions. The Cochrane *Q* test was used to evaluate the heterogeneity of each selected SNP. The MR‐Egger intercept test was conducted to detect horizontal pleiotropy, which was used to calculate the intercept term available after the linear regression analysis. The MR‐PRESSO examination assessed the total pleiotropy of the study to ensure a robust estimate. If there was significant heterogeneity between the SNPs, they were further analyzed using outlier‐corrected MR‐PRESSO. Funnel plots were used to assess possible directional pleiotropy. Forest plots were used to illustrate the effects of exposure and outcomes. Finally, leave‐one‐out sensitivity analysis was conducted to determine the stability of the results.

## 3 RESULTS

**Figure 2:**
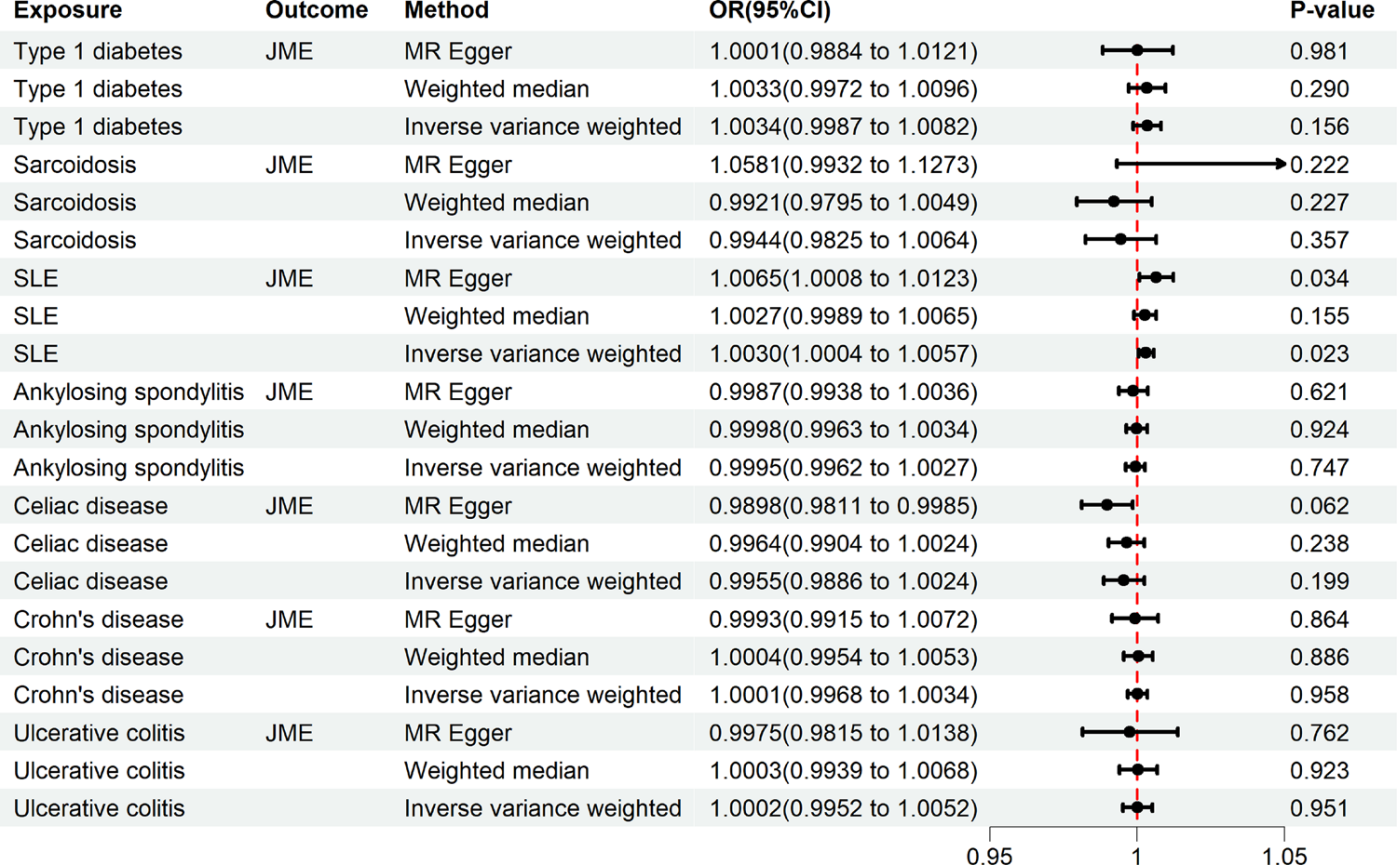
The results of the causal relationship between seven autoimmune diseases and JME using different MR methods. CI, confidence interval; JME, juvenile myoclonic epilepsy; MR, Mendelian randomization; OR, odds ratio; SLE, systemic lupus erythematosus. [Color figure can be viewed at wileyonlinelibrary.com]

**Figure 3:**
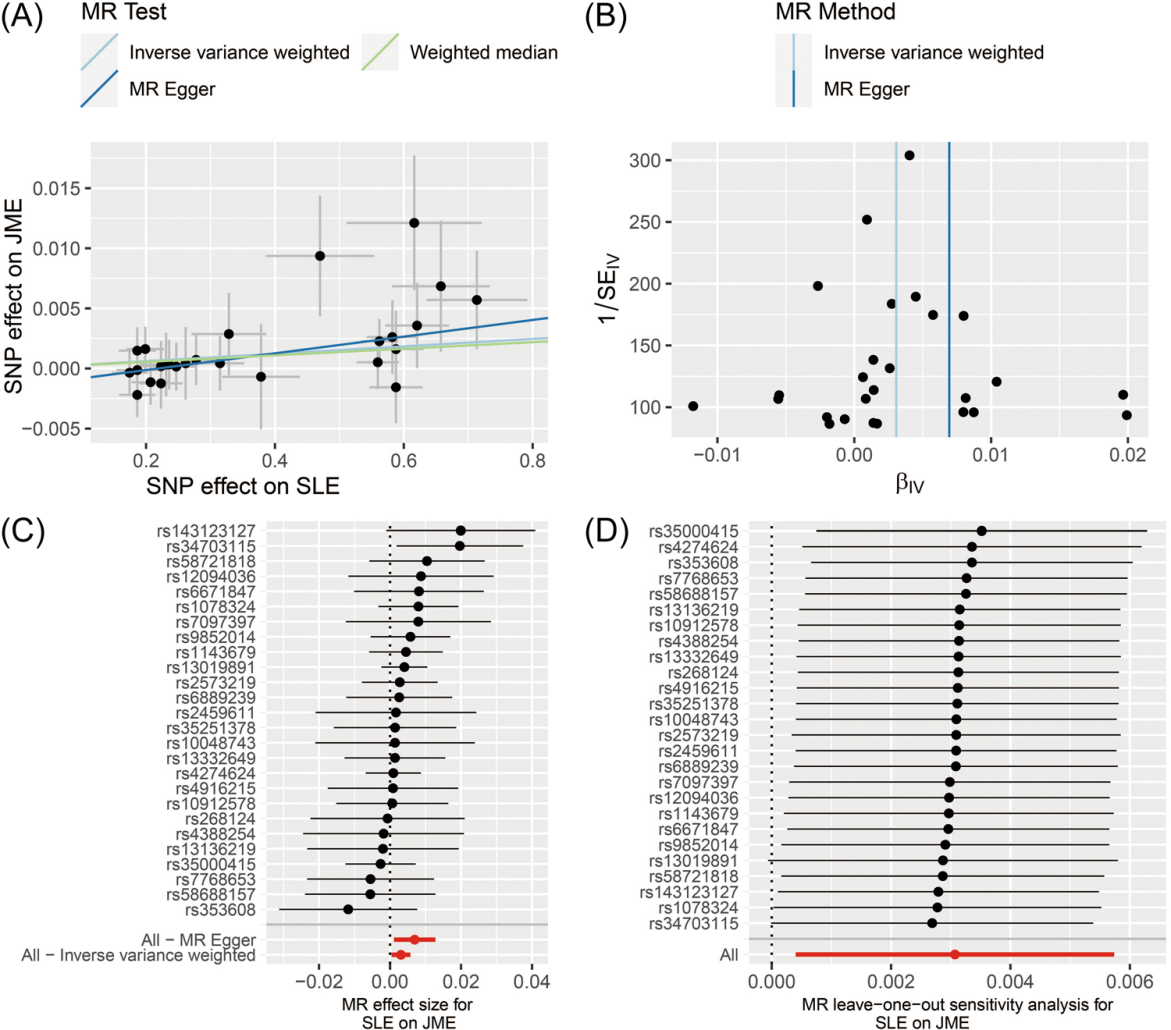
MR of the causal effect of SLE and risk of JME. (A) Scatter plot of potential effects on SLE to JME. The slope of each line correlates with the estimated Mendelian randomization effect among three different methods, including the IVW method, MR‐Egger, and Weighted median. (B) Funnel plot of potential effects on SLE to JME. Vertical lines show the causal estimates using all SNPs combined into a single instrument for each of the two different methods (IVW random effects and MR‐Egger). (C) Forest plot of the causal relationship between SLE and JME. The causal effect of SLE on JME was estimated using each SNP singly using the Wald ratio. The two red lines show the combined causal estimate using all SNPs together in a single instrument, using each of the two different methods (IVW random effects and MR‐Egger). (D) MR leave‐one‐out sensitivity analysis for SLE on JME. Each black point represents the IVW MR method applied to estimate the causal effect of SLE on JME by excluding that particular variant from the analysis. The red point depicts the IVW estimate using all SNPs. There are no instances where the exclusion of one particular SNP leads to dramatic changes in the overall result. IVW, inverse‐variance weighted; JME, juvenile myoclonic epilepsy; MR, Mendelian randomization; SLE, systemic lupus erythematosus; SNPs, single nucleotide polymorphisms. [Color figure can be viewed at wileyonlinelibrary.com]

The results of the univariable MR analysis are summarized in Figure 2. Finally, 28 SNPs were identified as instrumental variables in the SLE and JME analyses. Notably, genetic susceptibility to SLE was causally associated with an increased risk of JME (OR = 1.0030, 95% CI, 1.0004–1.0057; *p* = 0.023). However, no causal associations were found between the other SADs and JME (Supporting Information S1: Table S5). Subsequently, we performed a series of sensitivity analyses to evaluate any bias in MR assumptions. As for the causal association between SLE and JME, there was no evidence of heterogeneity in the IVW analysis (*p* = 0.969), and the MR‐Egger intercept suggested no horizontal pleiotropy (*p* = 0.192). Moreover, a global MR‐PRESSO test revealed no pleiotropy (Supporting Information S1: Table S9). The scatter plot and forest plot showed the effects of SLE on JME (Figure 3A,C). In addition, the funnel plot demonstrated no evidence of asymmetry, indicating a low risk of directional pleiotropy (Figure 3B). Leave‐one‐out analysis did not detect any significant outliers (Figure 3D). For confounding analysis, we manually investigated secondary traits (smoking, obesity, alcohol consumption, and diabetes) of the SNPs to validate the robustness of the results. After removing these two SNPs (rs597808 and rs6679677), the causality remained significant (OR = 1.0032, 95% CI, 1.0002–1.0064; *p* = 0.024). We performed a two‐step mediation MR analysis to assess the potential induction of JME in SLE via inflammatory cytokine‐mediating factors. The following inflammatory cytokines were included: interleukin (IL)‐1α, IL‐6, IL‐12, IL‐4, IL‐10, IL‐13, transforming growth factor (TGF) ‐β1, TGF ‐β2, TGF ‐β3, tumor necrosis factor (TNF)‐α, and type I interferon (IFN)‐γ. Our mediation analysis did not reveal a significant causal relationship between SLE and inflammatory cytokine levels (Supporting Information S1: Table S6). We did not observe a causal relationship between inflammatory cytokines and JME (Supporting Information S1: Table S7). Therefore, inflammatory cytokines did not mediate the relationship between SLE and JME. In the reverse analysis, no significant relationship was identified between genetic susceptibility to JME and autoimmune diseases (Supporting Information S1: Table S8).

## 4 DISCUSSION AND CONCLUSION

In this study, we provided new evidence for a causal relationship between genetic susceptibility to SLE and JME. Specifically, genetic susceptibility to SLE was causally associated with an increased risk of JME. However, there was almost no evidence in reverse MR analysis to suggest a causal genetic relationship between JME and SLE. Besides, there was no causal relationship between other autoimmune diseases (sarcoidosis, ankylosing spondylitis, T1DM, CD, UC, and celiac disease) and JME. Further subdivisions at the molecular level suggested that there was no causal relationship between SLE and inflammatory cytokines and between inflammatory cytokines and JME, revealing that inflammatory cytokines did not mediate the relationship between SLE and JME. However, elevated levels of inflammatory cytokines, autoantibodies in SADs, and antibodies against neuronal antigens may contribute to our understanding of the pathogenesis of autoimmune diseases and epilepsy.^3^, ^21^ Other unknown factors, the impact of different epilepsy comorbidities, and common risk factors (such as common etiologies or environmental triggering factors) may also explain this association.^3^, ^22^ The potential mechanisms underlying the association between SLE and JME remain largely unknown and may involve multiple mechanisms.

First, we speculated that antineuronal antibodies may play a crucial role in mediating the development of epilepsy in SLE. In a previous study, one‐tenth of a highly selected cohort of patients with epilepsy and SLE had serum‐containing antineuronal antibodies against known antigens, while a larger proportion also had antibodies against unknown neural cell antigens. The clinical significance of these antibodies remains unclear.^23^ Anti‐double stranded DNA (dsDNA) antibodies are markers of SLE. Further research has shown that high levels of anti‐dsDNA antibodies are present in the serum and cerebrospinal fluid of patients with Rasmussen encephalitis and other types of epilepsy.^24^, ^25^ Moreover, 14%–35% of patients with SLE have anti‐N‐methyl‐d‐aspartate (NMDA) NR2 antibodies, which are also present in those with several types of epilepsy. Some anti‐NMDA‐NR2 antibodies cross‐react with dsDNA, leading to inflammatory cascades and apoptosis in animal models, whereas others do not. However, most patients with epilepsy have only one type of antibody and oppose cross‐reactivity.^26^ Systemic antibodies (such as antiphospholipid antibodies) may directly affect cytokine production and neuronal excitability.^27^ Studies have shown that antiphospholipid antibodies can damage γ‐aminobutyric acid (GABA) receptor activity and induce depolarization of synaptic nervous systems, disrupting neuronal function by acting on nerve endings, which may have an impact on the related occurrence of epilepsy.^28^, ^29^ Some researchers believe that 20% of idiopathic adolescent epilepsy cases may be related to antiphospholipid antibodies.^28^ The influence of antiphospholipid antibodies (such as anticardiolipin, lupus anticoagulant, and anti‐β2‐glycoprotein I) on the coagulation cascade mediates the development of epilepsy in SLE.^27^, ^30^

Second, various inflammatory cytokines and immune complexes mediate neuronal damage and neuroinflammation, which are involved in the development of SLE and epilepsy.^31^, ^32^ SLE involves systemic inflammation and organ damage, and its onset is characterized by strong immune activation and widespread cell death.^33^ Both cause the release of extracellular high‐mobility group box 1 (HMGB1) and disordered IFN system‐mediated excessive IFN‐α production, contributing to the activation of adaptive immune response and the absence of cytoplastic HMGB protein with inter‐exposure to the secretion of IL‐1β.^34^, ^35^, ^36^ In addition, IL‐1β and HMGB1 enhanced the phosphorylation of NMDA receptor NR2B subunit, thereby increasing NMDA activity and the occurrence of epilepsy.^37^, ^38^ IL‐1β has been shown to combine with HMGB1 and enhance inflammatory activity, in which neuroinflammation plays a key role.^39^ Additionally, IL‐10 is associated with SLE.^40^ In the case of blood–brain barrier (BBB) injury after SLE vasculitis, IL‐10 crosses the BBB and increases the excitatory and inhibitory synaptic contacts in neurons.^41^ SLE upregulates the anti‐NMDA receptors in cerebrospinal fluid and upregulates circulating IL‐1β, IL‐8, IFN‐γ, and microglia‐activating antibodies.^42^, ^43^, ^44^ These key cytokines and cytokine receptors are involved in various mechanistic inflammatory pathways that lead to harmful synaptic changes and neuronal overexcitation, followed by seizures. Inflammatory factors not only regulate neural connections and excitability but also damage the BBB, thereby allowing further intrusion of harmful chemicals and mediators.^45^ Neuroinflammation is a sign of the onset of epilepsy and may be triggered not only by neuronal damage but also by immunological activation throughout the body, suggesting that neuroinflammation is a potential risk factor for SLE and epileptic diseases.^46^ Besides, it is reported that peripheral inflammation also increases susceptibility to seizures through cyclooxygenase‐2 ‐dependent microglial activation and upregulation of IL‐1β, IL‐6, and TNF‐α in the hippocampus, indicating that in addition to neuroinflammation, there is an association between peripheral inflammation and epilepsy.^47^

Third, genetic factors may be a common disease mechanism in both SLE and epilepsy. Recent genome‐wide linkage analyses revealed a common genetic polymorphism between SLE and epilepsy.^20^ Human leukocyte antigen (HLA) haplotypes may also be a common feature of these diseases, and shared HLA profiles may explain the association between seizures and autoimmune diseases. Different haplotypes have been reported in syndromes leading to autoimmune encephalitis and epilepsy.^48^, ^49^, ^50^

In other SADs, intestinal dysfunction and cross‐reactivity of systemic antibodies (such as anti‐glutamic acid decarboxylase antibodies) to neural antigens may be important mechanisms underlying seizures in patients with celiac disease.^2^ In T1DM, it must be considered that seizures may be the result of metabolic disorders, associated neuroinflammation/autoimmunity (anti‐glutamic acid decarboxylase antibodies), and genetic factors.^51^, ^52^ In conclusion, seizures in SLE and other SADs involve inflammatory cytokines and systemic autoantibodies. The shared HLA profile may also explain the association between seizures and SADs.

However, this study has several limitations. First, the participants were of European ancestry; therefore, the results should be carefully generalized to other populations. Second, the scale of impact was relatively small and should be interpreted with caution. Third, there are other possible unmeasured and residual confounding factors, such as those in many other epidemiological studies, which may have led to bias in the overall estimates. Fourth, the data in this study were from previous databases, and there is no first‐hand experimental evidence that could have impacted the existing research results. Fifth, genetic factors are not the only determining factors for the onset of SLE; environmental factors also play a role in this process. Therefore, our MR analysis did not show a categorical association between gene‐predicted SLE and JME risk; however, this does not rule out the potential impact of SLE on the pathophysiology of JME.

Therefore, further research on the relationship between SLE and JME and increased screening for seizure risk factors in patients with SLE and other autoimmune diseases is needed. The pathogenesis of SLE and JME may involve multiple mechanisms. Current research is limited, but the search for better biomarkers, including specific antibodies and cytokines that can be used to predict seizure risk and facilitate an analysis of the causal relationship between SLE and JME; this could potentially have important therapeutic implications, which may lead to the discovery of specific therapies that target antibodies or inflammatory responses. Future database studies are needed to clarify whether an association between SLE and JME is present in all SADs, which may require large‐scale data. In summary, this study may provide guidance for screening risk factors for seizures and exploring potential treatments in SLE and JME, and even all SADs and JME.

## AUTHOR CONTRIBUTIONS

Sirui Chen and Ningning Zhang wrote and edited the original manuscript. Ruirui Zhang and Lan Zhang contributed to collecting literature. Junqiang Li, Yaqing Liu, and Dadong Luo were involved in image drawing and editing. Yunan Wang and Xinyue Duan were involved in the article revision. Xin Tian and Tiancheng Wang were in charge of the establishment of the overall idea, article revision, and supervision. Tiancheng Wang was in charge of financial support and final review.

## ETHICS STATEMENT

Not applicable as this analysis used publicly available summary statistics that had already obtained ethical approval.

## CONFLICTS OF INTEREST STATEMENT

The authors declare no conflicts of interest.

## Supporting information

Supporting information.

## Data Availability

All data used in this study are publicly available, with relevant citations detailed.
