# Supplementary material for: The causal relationship between systemic lupus erythematosus and juvenile myoclonic epilepsy: A Mendelian randomization study and mediation analysis
Source: Ibrain. 2025 Jan 7;11(1):98–105. doi: 10.1002/ibra.12191 (PMC11911104; doi:10.1002/ibra.12191)
Supplement: Supplementary file 1 — Supporting information. [file IBRA-11-98-s001.docx]

**Supplementary Material**

Supplementary Table S1 Sources of exposure

| **Exposure** | **First Author (Year)** | **Sample Size** | **Population** | **MRC-IEU id:** |
| --- | --- | --- | --- | --- |
| Ankylosing spondylitis | NA(2021) | 166144 | European | finn-b-M13_ANKYLOSPON |
| Celiac disease | Trynka(2011) | 24269 | European | ieu-a-1058 |
| Crohn’s disease | Liu(2015) | 20883 | European | ieu-a-30 |
| Sarcoidosis | NA(2021) | 217758 | European | finn-b-D3_SARCOIDOSIS |
| Systemic lupus erythematosus | Bentham J(2015) | 14267 | European | ebi-a-GCST003156 |
| Type 1 diabetes mellitus | Forgetta V(2020) | 24840 | European | ebi-a-GCST010681 |
| Ulcerative colitis | Liu(2015) | 27432 | European | ieu-a-32 |

Supplementary Table S2 Sources of Mediatiors

| **Exposure** | **First Author (Year)** | **Sample Size** | **Population** | **MRC-IEU id:** |
| --- | --- | --- | --- | --- |
| IL-1α | Suhre K (2019) | 3080 | European | prot-c-4851_25_1 |
| IL-6 | Suhre K (2019) | 3080 | European | prot-c-4673_13_2 |
| IL-12 | Suhre K (2019) | 3080 | European | prot-c-2455_17_4 |
| IL-4 | Suhre K (2019) | 3080 | European | prot-c-2906_55_3 |
| IL-10 | Suhre K (2019) | 3080 | European | prot-c-2773_50_2 |
| IL-13 | Suhre K (2019) | 3080 | European | prot-c-3072_4_2 |
| TGF-β1 | Suhre K (2019) | 3080 | European | prot-c-2333_72_1 |
| TGF-β2 | Suhre K (2019) | 3080 | European | prot-c-4156_74_1 |
| TGF-β3 | Suhre K (2019) | 3080 | European | prot-c-3520_58_1 |
| TNF-α | Suhre K (2019) | 3080 | European | prot-c-3722_49_2 |
| IFN-γ | Suhre K (2019) | 3080 | European | prot-c-2989_17_2 |

Supplementary Table S3 Instrumental variables for autoimmune diseases

| Exposures | SNP | beta | eaf | se | pval | F |
| --- | --- | --- | --- | --- | --- | --- |
| AS | rs112733823 | 0.36 | 0.2165 | 0.0468 | 1.43E-14 | 59.172 |
| AS | rs13033284 | -0.221 | 0.6277 | 0.0386 | 9.67E-09 | 32.899 |
| AS | rs16894011 | 2.108 | 0.07267 | 0.0886 | 5.32E-125 | 565.806 |
| AS | rs62394289 | 0.374 | 0.1376 | 0.0557 | 1.90E-11 | 45.037 |
| AS | rs76644067 | 0.733 | 0.04745 | 0.0946 | 9.44E-15 | 60.005 |
| AS | rs9378220 | -0.693 | 0.2269 | 0.0558 | 1.97E-35 | 154.374 |
| AS | rs10752747 | -0.116 | 0.3088 | 0.0198763 | 5.05E-09 | 34.176 |
| Celiac disease | rs12527282 | -0.155 | 0.2799 | 0.0209646 | 1.69E-13 | 54.346 |
| Celiac disease | rs130078 | -0.577 | 0.7809 | 0.0242572 | 3.35E-125 | 566.441 |
| Celiac disease | rs13030124 | -0.105 | 0.4502 | 0.018842 | 2.40E-08 | 31.136 |
| Celiac disease | rs13119723 | -0.309 | 0.1534 | 0.0277233 | 7.60E-29 | 124.1 |
| Celiac disease | rs13198474 | 0.946 | 0.05478 | 0.0311878 | 1.00E-200 | 920.516 |
| Celiac disease | rs2441467 | 0.105 | 0.4183 | 0.0186632 | 1.70E-08 | 31.81 |
| Celiac disease | rs7162232 | -0.117 | 0.6813 | 0.0202624 | 7.97E-09 | 33.281 |
| Crohn's disease | rs1056441 | 0.167 | 0.697697 | 0.0255 | 5.44E-11 | 42.89 |
| Crohn's disease | rs10761659 | 0.212 | 0.55269 | 0.0237 | 3.42E-19 | 80.02 |
| Crohn's disease | rs11209026 | -0.995 | 0.056321 | 0.0639 | 1.05E-54 | 242.559 |
| Crohn's disease | rs11236797 | 0.181 | 0.473264 | 0.0231 | 4.85E-15 | 61.466 |
| Crohn's disease | rs112401990 | 0.132 | 0.373412 | 0.0237 | 2.35E-08 | 31.116 |
| Crohn's disease | rs11564236 | 0.519 | 0.034419 | 0.0595 | 2.85E-18 | 76.113 |
| Crohn's disease | rs12194825 | -0.172 | 0.18587 | 0.0298 | 8.00E-09 | 33.277 |
| Crohn's disease | rs1250573 | -0.171 | 0.287447 | 0.0264 | 9.01E-11 | 41.904 |
| Crohn's disease | rs12717899 | 0.159 | 0.793845 | 0.0289 | 3.59E-08 | 30.344 |
| Crohn's disease | rs1297271 | -0.155 | 0.429877 | 0.0237 | 6.28E-11 | 42.718 |
| Crohn's disease | rs1332099 | -0.212 | 0.515025 | 0.0231 | 4.36E-20 | 83.906 |
| Crohn's disease | rs140054334 | 0.35 | 0.038307 | 0.0628 | 2.57E-08 | 31.025 |
| Crohn's disease | rs1456896 | 0.139 | 0.697556 | 0.0251 | 2.90E-08 | 30.801 |
| Crohn's disease | rs147018773 | 0.322 | 0.096698 | 0.0375 | 8.89E-18 | 73.593 |
| Crohn's disease | rs147684209 | 0.155 | 0.369415 | 0.0244 | 2.34E-10 | 40.302 |
| Crohn's disease | rs151314883 | -0.224 | 0.157585 | 0.0327 | 7.12E-12 | 46.922 |
| Crohn's disease | rs1873625 | 0.181 | 0.320264 | 0.0243 | 1.09E-13 | 55.3 |
| Crohn's disease | rs1932990 | 0.153 | 0.253841 | 0.0263 | 6.02E-09 | 33.799 |
| Crohn's disease | rs2076756 | 0.4 | 0.283672 | 0.0242 | 3.24E-61 | 272.94 |
| Crohn's disease | rs2188962 | 0.212 | 0.439546 | 0.0228 | 1.36E-20 | 86.782 |
| Crohn's disease | rs2505640 | -0.146 | 0.643588 | 0.0237 | 7.61E-10 | 37.794 |
| Crohn's disease | rs281379 | 0.14 | 0.489275 | 0.0238 | 4.26E-09 | 34.502 |
| Crohn's disease | rs3024505 | 0.178 | 0.16156 | 0.0302 | 3.90E-09 | 34.702 |
| Crohn's disease | rs3091315 | -0.18 | 0.265588 | 0.0263 | 9.52E-12 | 46.582 |
| Crohn's disease | rs3810936 | 0.208 | 0.698408 | 0.0263 | 2.46E-15 | 62.427 |
| Crohn's disease | rs4077515 | 0.216 | 0.419834 | 0.0235 | 4.37E-20 | 84.406 |
| Crohn's disease | rs444210 | 0.163 | 0.546838 | 0.0229 | 1.02E-12 | 50.915 |
| Crohn's disease | rs4851586 | -0.169 | 0.759729 | 0.0261 | 9.94E-11 | 41.877 |
| Crohn's disease | rs4902642 | -0.129 | 0.409159 | 0.0236 | 4.34E-08 | 29.97 |
| Crohn's disease | rs4921497 | 0.16 | 0.329415 | 0.0244 | 5.49E-11 | 43.16 |
| Crohn's disease | rs56062135 | 0.193 | 0.234271 | 0.0269 | 7.45E-13 | 51.528 |
| Crohn's disease | rs6588243 | 0.132 | 0.589982 | 0.0234 | 1.78E-08 | 31.679 |
| Crohn's disease | rs6704109 | 0.202 | 0.256123 | 0.0256 | 2.77E-15 | 62.263 |
| Crohn's disease | rs6873866 | -0.168 | 0.534873 | 0.0239 | 2.06E-12 | 49.467 |
| Crohn's disease | rs697693 | 0.172 | 0.201415 | 0.0281 | 8.36E-10 | 37.596 |
| Crohn's disease | rs7276302 | -0.172 | 0.608306 | 0.0231 | 1.23E-13 | 55.182 |
| Crohn's disease | rs72798422 | 0.59 | 0.047701 | 0.0508 | 3.19E-31 | 135.068 |
| Crohn's disease | rs744166 | -0.129 | 0.408159 | 0.0233 | 2.92E-08 | 30.795 |
| Crohn's disease | rs7543234 | 0.155 | 0.238986 | 0.0267 | 6.10E-09 | 33.918 |
| Crohn's disease | rs7713270 | 0.297 | 0.623824 | 0.0241 | 6.97E-35 | 151.466 |
| Crohn's disease | rs78487399 | 0.226 | 0.100704 | 0.037 | 1.03E-09 | 37.276 |
| Crohn's disease | rs80262450 | 0.283 | 0.112701 | 0.0353 | 1.08E-15 | 64.319 |
| Crohn's disease | rs8137950 | 0.174 | 0.199556 | 0.0286 | 1.17E-09 | 37.012 |
| Crohn's disease | rs907092 | 0.13 | 0.471412 | 0.0228 | 1.01E-08 | 32.708 |
| Crohn's disease | rs921720 | 0.163 | 0.618841 | 0.0237 | 6.40E-12 | 47.241 |
| Multiple sclerosis | rs11129295 | 0.109 | NA | 0.0191318 | 1.14E-08 | 32.586 |
| Multiple sclerosis | rs11154801 | 0.137 | NA | 0.0193191 | 1.53E-12 | 50.015 |
| Multiple sclerosis | rs11581062 | 0.125 | NA | 0.0200179 | 3.74E-10 | 39.241 |
| Multiple sclerosis | rs12746893 | 0.141 | NA | 0.0203783 | 4.18E-12 | 48.037 |
| Multiple sclerosis | rs1335532 | -0.163 | NA | 0.027236 | 2.01E-09 | 35.968 |
| Multiple sclerosis | rs17066096 | 0.128 | NA | 0.0203511 | 3.41E-10 | 39.421 |
| Multiple sclerosis | rs1738074 | 0.124 | NA | 0.0188726 | 5.27E-11 | 43.075 |
| Multiple sclerosis | rs1800693 | 0.111 | NA | 0.017442 | 1.84E-10 | 40.634 |
| Multiple sclerosis | rs2119704 | -0.233 | NA | 0.0371935 | 3.46E-10 | 39.395 |
| Multiple sclerosis | rs2248359 | -0.112 | NA | 0.0191405 | 5.15E-09 | 34.133 |
| Multiple sclerosis | rs2293370 | -0.15 | NA | 0.0246021 | 1.08E-09 | 37.178 |
| Multiple sclerosis | rs3118470 | 0.116 | NA | 0.0193084 | 2.05E-09 | 35.929 |
| Multiple sclerosis | rs4613763 | 0.194 | NA | 0.0258561 | 6.86E-14 | 56.107 |
| Multiple sclerosis | rs4648356 | -0.149 | NA | 0.0196774 | 3.10E-14 | 57.668 |
| Multiple sclerosis | rs4902647 | -0.103 | NA | 0.018734 | 3.81E-08 | 30.246 |
| Multiple sclerosis | rs650258 | 0.112 | NA | 0.0186117 | 1.74E-09 | 36.245 |
| Multiple sclerosis | rs669607 | 0.121 | NA | 0.0181523 | 2.93E-11 | 44.222 |
| Multiple sclerosis | rs7200786 | -0.143 | NA | 0.0190472 | 6.30E-14 | 56.276 |
| Multiple sclerosis | rs771767 | -0.113 | NA | 0.0196377 | 1.00E-08 | 32.833 |
| Multiple sclerosis | rs874628 | -0.114 | NA | 0.0207837 | 4.32E-08 | 30.002 |
| Multiple sclerosis | rs9282641 | -0.191 | NA | 0.0315494 | 1.54E-09 | 36.48 |
| Sarcoidosis | rs1079242 | -0.257 | 0.5595 | 0.0328 | 4.06E-15 | 61.536 |
| Sarcoidosis | rs11690163 | 0.189 | 0.5999 | 0.0331 | 1.08E-08 | 32.742 |
| Sarcoidosis | rs479777 | -0.183 | 0.3838 | 0.0333 | 3.80E-08 | 30.3 |
| Sarcoidosis | rs7566220 | 0.208 | 0.2791 | 0.0362 | 8.98E-09 | 33.078 |
| SLE | rs10048743 | -0.231 | NA | 0.0412056 | 2.04E-08 | 31.458 |
| SLE | rs1078324 | -0.713 | NA | 0.0781665 | 7.11E-20 | 83.284 |
| SLE | rs10912578 | -0.247 | NA | 0.0309918 | 1.65E-15 | 63.447 |
| SLE | rs1143679 | 0.582 | NA | 0.0399866 | 5.03E-48 | 212.002 |
| SLE | rs12094036 | -0.329 | NA | 0.0578595 | 1.37E-08 | 32.235 |
| SLE | rs13019891 | -0.562 | NA | 0.0290336 | 1.65E-83 | 374.848 |
| SLE | rs13136219 | -0.174 | NA | 0.027787 | 3.50E-10 | 39.371 |
| SLE | rs13332649 | -0.315 | NA | 0.0375683 | 5.43E-17 | 70.175 |
| SLE | rs143123127 | 0.47 | NA | 0.0840342 | 2.23E-08 | 31.282 |
| SLE | rs2459611 | 0.261 | NA | 0.045245 | 7.62E-09 | 33.37 |
| SLE | rs2573219 | 0.588 | NA | 0.0429292 | 1.13E-42 | 187.471 |
| SLE | rs268124 | 0.186 | NA | 0.0323703 | 8.60E-09 | 33.134 |
| SLE | rs34703115 | -0.616 | NA | 0.104778 | 4.08E-09 | 34.585 |
| SLE | rs35000415 | 0.588 | NA | 0.041539 | 1.86E-45 | 200.229 |
| SLE | rs35251378 | -0.236 | NA | 0.0324266 | 3.61E-13 | 52.844 |
| SLE | rs353608 | 0.186 | NA | 0.0280198 | 2.93E-11 | 44.222 |
| SLE | rs4274624 | -0.56 | NA | 0.0326791 | 9.73E-66 | 293.251 |
| SLE | rs4388254 | 0.378 | NA | 0.0603977 | 3.71E-10 | 39.259 |
| SLE | rs4916215 | 0.223 | NA | 0.0339693 | 5.07E-11 | 43.152 |
| SLE | rs58688157 | -0.223 | NA | 0.0335647 | 2.97E-11 | 44.198 |
| SLE | rs58721818 | 0.658 | NA | 0.0755941 | 3.38E-18 | 75.656 |
| SLE | rs597808 | -0.163 | NA | 0.0294736 | 3.51E-08 | 30.405 |
| SLE | rs6671847 | 0.199 | NA | 0.0289651 | 6.64E-12 | 47.131 |
| SLE | rs6679677 | 0.336 | NA | 0.0464854 | 4.55E-13 | 52.392 |
| SLE | rs6889239 | 0.278 | NA | 0.03174 | 2.19E-18 | 76.511 |
| SLE | rs7097397 | -0.186 | NA | 0.0287118 | 8.60E-11 | 42.116 |
| SLE | rs7768653 | -0.207 | NA | 0.0296891 | 3.11E-12 | 48.619 |
| SLE | rs9852014 | 0.621 | NA | 0.0492727 | 2.26E-36 | 158.628 |
| Type 1 diabetes mellitus | rs10183097 | 0.205 | 1.36E-01 | 0.0322 | 1.82E-10 | 40.651 |
| Type 1 diabetes mellitus | rs10760335 | 0.136 | 3.21E-01 | 0.0243 | 2.43E-08 | 31.185 |
| Type 1 diabetes mellitus | rs10774624 | -0.256 | 5.04E-01 | 0.0244 | 1.34E-25 | 109.734 |
| Type 1 diabetes mellitus | rs10830227 | 0.158 | 5.74E-01 | 0.0233 | 1.02E-11 | 46.1 |
| Type 1 diabetes mellitus | rs10865468 | -0.162 | 2.53E-01 | 0.0277 | 4.66E-09 | 34.373 |
| Type 1 diabetes mellitus | rs10911399 | -0.371 | 4.55E-02 | 0.064 | 6.75E-09 | 33.549 |
| Type 1 diabetes mellitus | rs1131017 | -0.246 | 5.80E-01 | 0.0238 | 4.24E-25 | 106.923 |
| Type 1 diabetes mellitus | rs11571297 | -0.196 | 4.84E-01 | 0.0237 | 1.11E-16 | 68.673 |
| Type 1 diabetes mellitus | rs12722495 | -0.315 | 1.12E-01 | 0.0408 | 1.27E-14 | 59.418 |
| Type 1 diabetes mellitus | rs13182737 | 0.147 | 2.55E-01 | 0.0259 | 1.49E-08 | 31.995 |
| Type 1 diabetes mellitus | rs17125653 | 0.236 | 7.70E-02 | 0.0402 | 4.75E-09 | 34.319 |
| Type 1 diabetes mellitus | rs1869449 | 0.177 | 2.97E-01 | 0.0269 | 4.55E-11 | 43.247 |
| Type 1 diabetes mellitus | rs192324744 | 0.562 | 1.31E-02 | 0.0875 | 1.36E-10 | 41.253 |
| Type 1 diabetes mellitus | rs194749 | -0.164 | 2.46E-01 | 0.0281 | 5.37E-09 | 33.979 |
| Type 1 diabetes mellitus | rs202520 | -0.157 | 7.22E-01 | 0.0256 | 7.97E-10 | 37.755 |
| Type 1 diabetes mellitus | rs2071647 | 0.153 | 2.76E-01 | 0.0258 | 3.30E-09 | 34.984 |
| Type 1 diabetes mellitus | rs2269247 | 0.171 | 1.80E-01 | 0.0295 | 7.28E-09 | 33.561 |
| Type 1 diabetes mellitus | rs4566101 | 0.176 | 2.70E-01 | 0.0255 | 6.23E-12 | 47.367 |
| Type 1 diabetes mellitus | rs59680223 | 0.642 | 8.10E-03 | 0.1032 | 5.00E-10 | 38.712 |
| Type 1 diabetes mellitus | rs62410259 | -0.38 | 7.70E-02 | 0.0533 | 1.02E-12 | 50.722 |
| Type 1 diabetes mellitus | rs6679677 | 0.653 | 9.93E-02 | 0.0346 | 3.42E-79 | 355.857 |
| Type 1 diabetes mellitus | rs6719660 | 0.292 | 9.35E-01 | 0.0524 | 2.52E-08 | 31.01 |
| Type 1 diabetes mellitus | rs741172 | -0.203 | 3.21E-01 | 0.0258 | 3.11E-15 | 62.153 |
| Type 1 diabetes mellitus | rs8056814 | 0.264 | 7.93E-02 | 0.0415 | 1.99E-10 | 40.499 |
| Ulcerative colitis | rs10182512 | 0.161 | 3.50E-01 | 0.0223 | 5.19E-13 | 51.995 |
| Ulcerative colitis | rs10272963 | -0.172 | 4.26E-01 | 0.0216 | 1.69E-15 | 63.338 |
| Ulcerative colitis | rs10737481 | 0.25 | 5.56E-01 | 0.0216 | 4.37E-31 | 134.071 |
| Ulcerative colitis | rs11209026 | -0.562 | 5.89E-02 | 0.0517 | 1.58E-27 | 118.039 |
| Ulcerative colitis | rs12612675 | 0.123 | 4.03E-01 | 0.0219 | 1.98E-08 | 31.494 |
| Ulcerative colitis | rs12817473 | 0.191 | 3.82E-01 | 0.0217 | 1.71E-18 | 77.23 |
| Ulcerative colitis | rs137845 | 0.118 | 5.15E-01 | 0.0212 | 2.38E-08 | 31.085 |
| Ulcerative colitis | rs1801274 | -0.183 | 4.83E-01 | 0.0217 | 3.78E-17 | 71.038 |
| Ulcerative colitis | rs1886731 | -0.141 | 4.81E-01 | 0.0221 | 2.25E-10 | 40.418 |
| Ulcerative colitis | rs2212434 | 0.142 | 4.60E-01 | 0.0213 | 2.46E-11 | 44.381 |
| Ulcerative colitis | rs254559 | 0.124 | 4.04E-01 | 0.0215 | 7.63E-09 | 33.427 |
| Ulcerative colitis | rs3024493 | 0.236 | 1.68E-01 | 0.0276 | 1.09E-17 | 73.299 |
| Ulcerative colitis | rs35730213 | -0.167 | 2.67E-01 | 0.0245 | 8.81E-12 | 46.462 |
| Ulcerative colitis | rs3829111 | 0.156 | 4.17E-01 | 0.0214 | 2.89E-13 | 53.347 |
| Ulcerative colitis | rs483905 | 0.129 | 2.94E-01 | 0.0228 | 1.57E-08 | 31.964 |
| Ulcerative colitis | rs484356 | -0.134 | 3.28E-01 | 0.0228 | 3.95E-09 | 34.644 |
| Ulcerative colitis | rs6933404 | 0.167 | 2.16E-01 | 0.0252 | 3.68E-11 | 43.811 |
| Ulcerative colitis | rs7282490 | -0.14 | 6.04E-01 | 0.0214 | 7.08E-11 | 42.615 |
| Ulcerative colitis | rs7523335 | -0.17 | 1.77E-01 | 0.0285 | 2.29E-09 | 35.747 |
| Ulcerative colitis | rs7752873 | 0.182 | 1.37E-01 | 0.0303 | 1.83E-09 | 36.197 |
| Ulcerative colitis | rs7911680 | -0.172 | 4.90E-01 | 0.0213 | 8.27E-16 | 65.056 |
| Ulcerative colitis | rs798502 | -0.136 | 2.83E-01 | 0.0239 | 1.21E-08 | 32.617 |
| Ulcerative colitis | rs9823546 | 0.177 | 3.10E-01 | 0.0223 | 2.29E-15 | 62.928 |
| Ulcerative colitis | rs989960 | -0.129 | 4.25E-01 | 0.0215 | 1.77E-09 | 36.054 |
| Ulcerative colitis | rs9977672 | -0.245 | 2.51E-01 | 0.0261 | 6.21E-21 | 88.12 |

SNP: single nucleotide polymorphism; EAF: effect allele frequency; Beta was obtained by allele-related effects; SE: standard error; F: F-statistics；AS：ankylosing spondylitis；SLE: systemic lupus erythematosus.

Supplementary Table S4 Instrumental variables for juvenile myoclonic epilepsy

| Outcomes | SNP | beta | eaf | se | pval | F |
| --- | --- | --- | --- | --- | --- | --- |
| AS | rs1046276 | -0.013 | 0.648 | 0.001925 | 2.5E-11 | 44.496 |
| AS | rs1077077 | -0.009 | 0.645 | 0.001913 | 4.3E-06 | 21.113 |
| AS | rs11650615 | -0.01 | 0.286 | 0.002041 | 1.6E-06 | 23.057 |
| AS | rs118100612 | 0.048 | 0.011 | 0.008923 | 7.2E-08 | 29.016 |
| AS | rs12652623 | 0.018 | 0.061 | 0.00385 | 4.5E-06 | 21.049 |
| AS | rs1497633 | 0.01 | 0.626 | 0.001913 | 6.2E-08 | 29.305 |
| AS | rs16877398 | 0.021 | 0.048 | 0.004275 | 7.8E-07 | 24.408 |
| AS | rs17125318 | 0.015 | 0.094 | 0.003201 | 4.0E-06 | 21.25 |
| AS | rs17631826 | 0.024 | 0.032 | 0.005291 | 4.6E-06 | 21.01 |
| AS | rs36009174 | -0.009 | 0.542 | 0.001854 | 1.7E-06 | 22.931 |
| AS | rs4390729 | 0.012 | 0.773 | 0.002193 | 1.4E-07 | 27.709 |
| AS | rs456605 | 0.01 | 0.573 | 0.00189 | 2.4E-07 | 26.674 |
| AS | rs4956002 | 0.013 | 0.864 | 0.002711 | 3.7E-06 | 21.436 |
| AS | rs6107905 | -0.012 | 0.809 | 0.002353 | 7.4E-07 | 24.507 |
| AS | rs6439158 | -0.014 | 0.896 | 0.003014 | 3.6E-06 | 21.452 |
| AS | rs6662502 | 0.009 | 0.344 | 0.001937 | 3.1E-06 | 21.767 |
| AS | rs7953238 | -0.01 | 0.754 | 0.002148 | 2.9E-06 | 21.903 |
| AS | rs8092661 | 0.009 | 0.375 | 0.00195 | 2.5E-06 | 22.159 |
| AS | rs954415 | -0.011 | 0.711 | 0.002045 | 2.4E-07 | 26.702 |
| AS | rs9615811 | 0.011 | 0.236 | 0.00217 | 2.6E-07 | 26.523 |
| Crohn's disease | rs1046276 | -0.013 | 0.648 | 0.001925 | 2.5E-11 | 44.496 |
| Crohn's disease | rs1077077 | -0.009 | 0.645 | 0.001913 | 4.3E-06 | 21.113 |
| Crohn's disease | rs11650615 | -0.01 | 0.286 | 0.002041 | 1.6E-06 | 23.057 |
| Crohn's disease | rs118100612 | 0.048 | 0.011 | 0.008923 | 7.2E-08 | 29.016 |
| Crohn's disease | rs12652623 | 0.018 | 0.061 | 0.00385 | 4.5E-06 | 21.049 |
| Crohn's disease | rs1497633 | 0.01 | 0.626 | 0.001913 | 6.2E-08 | 29.305 |
| Crohn's disease | rs16877398 | 0.021 | 0.048 | 0.004275 | 7.8E-07 | 24.408 |
| Crohn's disease | rs17125318 | 0.015 | 0.094 | 0.003201 | 4.0E-06 | 21.25 |
| Crohn's disease | rs17631826 | 0.024 | 0.032 | 0.005291 | 4.6E-06 | 21.01 |
| Crohn's disease | rs36009174 | -0.009 | 0.542 | 0.001854 | 1.7E-06 | 22.931 |
| Crohn's disease | rs4390729 | 0.012 | 0.773 | 0.002193 | 1.4E-07 | 27.709 |
| Crohn's disease | rs456605 | 0.01 | 0.573 | 0.00189 | 2.4E-07 | 26.674 |
| Crohn's disease | rs4956002 | 0.013 | 0.864 | 0.002711 | 3.7E-06 | 21.436 |
| Crohn's disease | rs6107905 | -0.012 | 0.809 | 0.002353 | 7.4E-07 | 24.507 |
| Crohn's disease | rs6439158 | -0.014 | 0.896 | 0.003014 | 3.6E-06 | 21.452 |
| Crohn's disease | rs6662502 | 0.009 | 0.344 | 0.001937 | 3.1E-06 | 21.767 |
| Crohn's disease | rs7953238 | -0.01 | 0.754 | 0.002148 | 2.9E-06 | 21.903 |
| Crohn's disease | rs8092661 | 0.009 | 0.375 | 0.00195 | 2.5E-06 | 22.159 |
| Crohn's disease | rs954415 | -0.011 | 0.711 | 0.002045 | 2.4E-07 | 26.702 |
| Crohn's disease | rs9615811 | 0.011 | 0.236 | 0.00217 | 2.6E-07 | 26.523 |
| Sarcoidosis | rs1046276 | -0.013 | 0.648 | 0.001925 | 2.5E-11 | 44.496 |
| Sarcoidosis | rs1077077 | -0.009 | 0.645 | 0.001913 | 4.3E-06 | 21.113 |
| Sarcoidosis | rs11650615 | -0.01 | 0.286 | 0.002041 | 1.6E-06 | 23.057 |
| Sarcoidosis | rs118100612 | 0.048 | 0.011 | 0.008923 | 7.2E-08 | 29.016 |
| Sarcoidosis | rs12652623 | 0.018 | 0.061 | 0.00385 | 4.5E-06 | 21.049 |
| Sarcoidosis | rs1497633 | 0.01 | 0.626 | 0.001913 | 6.2E-08 | 29.305 |
| Sarcoidosis | rs16877398 | 0.021 | 0.048 | 0.004275 | 7.8E-07 | 24.408 |
| Sarcoidosis | rs17125318 | 0.015 | 0.094 | 0.003201 | 4.0E-06 | 21.25 |
| Sarcoidosis | rs17631826 | 0.024 | 0.032 | 0.005291 | 4.6E-06 | 21.01 |
| Sarcoidosis | rs2395047 | 0.011 | 0.807 | 0.002322 | 4.9E-06 | 20.868 |
| Sarcoidosis | rs36009174 | -0.009 | 0.542 | 0.001854 | 1.7E-06 | 22.931 |
| Sarcoidosis | rs4390729 | 0.012 | 0.773 | 0.002193 | 1.4E-07 | 27.709 |
| Sarcoidosis | rs456605 | 0.01 | 0.573 | 0.00189 | 2.4E-07 | 26.674 |
| Sarcoidosis | rs4956002 | 0.013 | 0.864 | 0.002711 | 3.7E-06 | 21.436 |
| Sarcoidosis | rs6107905 | -0.012 | 0.809 | 0.002353 | 7.4E-07 | 24.507 |
| Sarcoidosis | rs6439158 | -0.014 | 0.896 | 0.003014 | 3.6E-06 | 21.452 |
| Sarcoidosis | rs6662502 | 0.009 | 0.344 | 0.001937 | 3.1E-06 | 21.767 |
| Sarcoidosis | rs7953238 | -0.01 | 0.754 | 0.002148 | 2.9E-06 | 21.903 |
| Sarcoidosis | rs8092661 | 0.009 | 0.375 | 0.00195 | 2.5E-06 | 22.159 |
| Sarcoidosis | rs954415 | -0.011 | 0.711 | 0.002045 | 2.4E-07 | 26.702 |
| Sarcoidosis | rs9615811 | 0.011 | 0.236 | 0.00217 | 2.6E-07 | 26.523 |
| SLE | rs1046276 | -0.013 | 0.648 | 0.001925 | 2.5E-11 | 44.496 |
| SLE | rs1077077 | -0.009 | 0.645 | 0.001913 | 4.3E-06 | 21.113 |
| SLE | rs118100612 | 0.048 | 0.011 | 0.008923 | 7.2E-08 | 29.016 |
| SLE | rs16877398 | 0.021 | 0.048 | 0.004275 | 7.8E-07 | 24.408 |
| SLE | rs17125318 | 0.015 | 0.094 | 0.003201 | 4.0E-06 | 21.25 |
| SLE | rs17631826 | 0.024 | 0.032 | 0.005291 | 4.6E-06 | 21.01 |
| SLE | rs2395047 | 0.011 | 0.807 | 0.002322 | 4.9E-06 | 20.868 |
| SLE | rs36009174 | -0.009 | 0.542 | 0.001854 | 1.7E-06 | 22.931 |
| SLE | rs4390729 | 0.012 | 0.773 | 0.002193 | 1.4E-07 | 27.709 |
| SLE | rs456605 | 0.01 | 0.573 | 0.00189 | 2.4E-07 | 26.674 |
| SLE | rs6439158 | -0.014 | 0.896 | 0.003014 | 3.6E-06 | 21.452 |
| SLE | rs6662502 | 0.009 | 0.344 | 0.001937 | 3.1E-06 | 21.767 |
| SLE | rs7953238 | -0.01 | 0.754 | 0.002148 | 2.9E-06 | 21.903 |
| SLE | rs8092661 | 0.009 | 0.375 | 0.00195 | 2.5E-06 | 22.159 |
| SLE | rs954415 | -0.011 | 0.711 | 0.002045 | 2.4E-07 | 26.702 |
| Type 1 diabetes mellitus | rs1046276 | -0.013 | 0.648 | 0.001925 | 2.5E-11 | 44.496 |
| Type 1 diabetes mellitus | rs1077077 | -0.009 | 0.645 | 0.001913 | 4.3E-06 | 21.113 |
| Type 1 diabetes mellitus | rs11650615 | -0.01 | 0.286 | 0.002041 | 1.6E-06 | 23.057 |
| Type 1 diabetes mellitus | rs118100612 | 0.048 | 0.011 | 0.008923 | 7.2E-08 | 29.016 |
| Type 1 diabetes mellitus | rs12652623 | 0.018 | 0.061 | 0.00385 | 4.5E-06 | 21.049 |
| Type 1 diabetes mellitus | rs1497633 | 0.01 | 0.626 | 0.001913 | 6.2E-08 | 29.305 |
| Type 1 diabetes mellitus | rs16877398 | 0.021 | 0.048 | 0.004275 | 7.8E-07 | 24.408 |
| Type 1 diabetes mellitus | rs17125318 | 0.015 | 0.094 | 0.003201 | 4.0E-06 | 21.25 |
| Type 1 diabetes mellitus | rs17631826 | 0.024 | 0.032 | 0.005291 | 4.6E-06 | 21.01 |
| Type 1 diabetes mellitus | rs2395047 | 0.011 | 0.807 | 0.002322 | 4.9E-06 | 20.868 |
| Type 1 diabetes mellitus | rs36009174 | -0.009 | 0.542 | 0.001854 | 1.7E-06 | 22.931 |
| Type 1 diabetes mellitus | rs4390729 | 0.012 | 0.773 | 0.002193 | 1.4E-07 | 27.709 |
| Type 1 diabetes mellitus | rs456605 | 0.01 | 0.573 | 0.00189 | 2.4E-07 | 26.674 |
| Type 1 diabetes mellitus | rs4956002 | 0.013 | 0.864 | 0.002711 | 3.7E-06 | 21.436 |
| Type 1 diabetes mellitus | rs6107905 | -0.012 | 0.809 | 0.002353 | 7.4E-07 | 24.507 |
| Type 1 diabetes mellitus | rs6439158 | -0.014 | 0.896 | 0.003014 | 3.6E-06 | 21.452 |
| Type 1 diabetes mellitus | rs6662502 | 0.009 | 0.344 | 0.001937 | 3.1E-06 | 21.767 |
| Type 1 diabetes mellitus | rs7953238 | -0.01 | 0.754 | 0.002148 | 2.9E-06 | 21.903 |
| Type 1 diabetes mellitus | rs8092661 | 0.009 | 0.375 | 0.00195 | 2.5E-06 | 22.159 |
| Type 1 diabetes mellitus | rs954415 | -0.011 | 0.711 | 0.002045 | 2.4E-07 | 26.702 |
| Type 1 diabetes mellitus | rs9615811 | 0.011 | 0.236 | 0.00217 | 2.6E-07 | 26.523 |
| Ulcerative colitis | rs1046276 | -0.013 | 0.648 | 0.001925 | 2.5E-11 | 44.496 |
| Ulcerative colitis | rs1077077 | -0.009 | 0.645 | 0.001913 | 4.3E-06 | 21.113 |
| Ulcerative colitis | rs11650615 | -0.01 | 0.286 | 0.002041 | 1.6E-06 | 23.057 |
| Ulcerative colitis | rs118100612 | 0.048 | 0.011 | 0.008923 | 7.2E-08 | 29.016 |
| Ulcerative colitis | rs12652623 | 0.018 | 0.061 | 0.00385 | 4.5E-06 | 21.049 |
| Ulcerative colitis | rs1497633 | 0.01 | 0.626 | 0.001913 | 6.2E-08 | 29.305 |
| Ulcerative colitis | rs16877398 | 0.021 | 0.048 | 0.004275 | 7.8E-07 | 24.408 |
| Ulcerative colitis | rs17125318 | 0.015 | 0.094 | 0.003201 | 4.0E-06 | 21.25 |
| Ulcerative colitis | rs17631826 | 0.024 | 0.032 | 0.005291 | 4.6E-06 | 21.01 |
| Ulcerative colitis | rs36009174 | -0.009 | 0.542 | 0.001854 | 1.7E-06 | 22.931 |
| Ulcerative colitis | rs4390729 | 0.012 | 0.773 | 0.002193 | 1.4E-07 | 27.709 |
| Ulcerative colitis | rs456605 | 0.01 | 0.573 | 0.00189 | 2.4E-07 | 26.674 |
| Ulcerative colitis | rs4956002 | 0.013 | 0.864 | 0.002711 | 3.7E-06 | 21.436 |
| Ulcerative colitis | rs6107905 | -0.012 | 0.809 | 0.002353 | 7.4E-07 | 24.507 |
| Ulcerative colitis | rs6439158 | -0.014 | 0.896 | 0.003014 | 3.6E-06 | 21.452 |
| Ulcerative colitis | rs6662502 | 0.009 | 0.344 | 0.001937 | 3.1E-06 | 21.767 |
| Ulcerative colitis | rs7953238 | -0.01 | 0.754 | 0.002148 | 2.9E-06 | 21.903 |
| Ulcerative colitis | rs8092661 | 0.009 | 0.375 | 0.00195 | 2.5E-06 | 22.159 |
| Ulcerative colitis | rs954415 | -0.011 | 0.711 | 0.002045 | 2.4E-07 | 26.702 |
| Ulcerative colitis | rs9615811 | 0.011 | 0.236 | 0.00217 | 2.6E-07 | 26.523 |
|  |  |  |  |  |  |  |

SNP: single nucleotide polymorphism; EAF: effect allele frequency; Beta was obtained by allele-related effects; SE: standard error; F: F-statistics; AS: ankylosing spondylitis；SLE: systemic lupus erythematosus.

Supplementary Table S5 Mendelian randomization analysis of autoimmune diseases with juvenile myoclonic epilepsy

| Exposures | Outcome | Method | Beta | P-value | OR | 95% CI  -low | 95% CI-up |
| --- | --- | --- | --- | --- | --- | --- | --- |
| AS | JME | MR Egger | -0.0013 | 0.621 | 0.9987 | 0.9938 | 1.0036 |
| AS | JME | Weighted median | -0.0002 | 0.924 | 0.9998 | 0.9963 | 1.0034 |
| AS | JME | Inverse variance weighted | -0.0005 | 0.747 | 0.9995 | 0.9962 | 1.0027 |
| AS | JME | Simple mode | -0.0013 | 0.663 | 0.9987 | 0.9931 | 1.0043 |
| AS | JME | Weighted mode | -0.0003 | 0.899 | 0.9997 | 0.9958 | 1.0037 |
| Celiac disease | JME | MR Egger | -0.0103 | 0.062 | 0.9898 | 0.9811 | 0.9985 |
| Celiac disease | JME | Weighted median | -0.0036 | 0.238 | 0.9964 | 0.9904 | 1.0024 |
| Celiac disease | JME | Inverse variance weighted | -0.0045 | 0.199 | 0.9955 | 0.9886 | 1.0024 |
| Celiac disease | JME | Simple mode | -0.005 | 0.334 | 0.995 | 0.9857 | 1.0045 |
| Celiac disease | JME | Weighted mode | -0.0063 | 0.056 | 0.9938 | 0.9884 | 0.9991 |
| Crohn's disease | JME | MR Egger | -0.0007 | 0.864 | 0.9993 | 0.9915 | 1.0072 |
| Crohn's disease | JME | Weighted median | 0.0004 | 0.886 | 1.0004 | 0.9954 | 1.0053 |
| Crohn's disease | JME | Inverse variance weighted | 1.00E-04 | 0.958 | 1.0001 | 0.9968 | 1.0034 |
| Crohn's disease | JME | Simple mode | 0.0012 | 0.805 | 1.0012 | 0.9919 | 1.0106 |
| Crohn's disease | JME | Weighted mode | -0.0006 | 0.851 | 0.9994 | 0.9932 | 1.0056 |
| Sarcoidosis | JME | MR Egger | 0.0565 | 0.222 | 1.0581 | 0.9932 | 1.1273 |
| Sarcoidosis | JME | Weighted median | -0.0079 | 0.227 | 0.9921 | 0.9795 | 1.0049 |
| Sarcoidosis | JME | Inverse variance weighted | -0.0056 | 0.357 | 0.9944 | 0.9825 | 1.0064 |
| Sarcoidosis | JME | Simple mode | -0.0154 | 0.245 | 0.9847 | 0.9643 | 1.0056 |
| Sarcoidosis | JME | Weighted mode | -0.0155 | 0.202 | 0.9846 | 0.9664 | 1.0032 |
| SLE | JME | MR Egger | 0.0065 | 0.034 | 1.0065 | 1.0008 | 1.0123 |
| SLE | JME | Weighted median | 0.0027 | 0.155 | 1.0027 | 0.999 | 1.0065 |
| SLE | JME | Inverse variance weighted | 0.003 | 0.022 | 1.0031 | 1.0004 | 1.0057 |
| SLE | JME | Simple mode | 0.0013 | 0.681 | 1.0013 | 0.9951 | 1.0077 |
| SLE | JME | Weighted mode | 0.0027 | 0.268 | 1.0026 | 0.998 | 1.0074 |
| Type 1 diabetes mellitus | JME | MR Egger | 0.0001 | 0.981 | 1.0001 | 0.9884 | 1.0121 |
| Type 1 diabetes mellitus | JME | Weighted median | 0.0033 | 0.29 | 1.0033 | 0.9972 | 1.0096 |
| Type 1 diabetes mellitus | JME | Inverse variance weighted | 0.0034 | 0.156 | 1.0034 | 0.9987 | 1.0082 |
| Type 1 diabetes mellitus | JME | Simple mode | 0.0148 | 0.042 | 1.0149 | 1.0013 | 1.0286 |
| Type 1 diabetes mellitus | JME | Weighted mode | 0.0016 | 0.715 | 1.0016 | 0.9932 | 1.01 |
| Ulcerative colitis | JME | MR Egger | -0.0025 | 0.762 | 0.9975 | 0.9815 | 1.0138 |
| Ulcerative colitis | JME | Weighted median | 0.0003 | 0.923 | 1.0003 | 0.9939 | 1.0068 |
| Ulcerative colitis | JME | Inverse variance weighted | 0.0002 | 0.951 | 1.0002 | 0.9952 | 1.0052 |
| Ulcerative colitis | JME | Simple mode | -0.0004 | 0.941 | 0.9996 | 0.9889 | 1.0104 |
| Ulcerative colitis | JME | Weighted mode | 0.0002 | 0.972 | 1.0002 | 0.9915 | 1.0089 |

The results of MR were shown by approach of MR-Egger, Weighted median, Inverse variance weighted, Simple mode and Weighted mode. AS: ankylosing spondylitis; JME: juvenile myoclonic epilepsy; SLE: systemic lupus erythematosus; CI: confidence intervals; OR: odds ratio; MR, Mendelian randomization

Supplementary Table S6 Mendelian Randomization analysis of systemic lupus erythematosus and inflammatory cytokines

| Exposures | Outcome | Method | Beta | P-value | OR | 95% CI-low | 95% CI-up |
| --- | --- | --- | --- | --- | --- | --- | --- |
| SLE | TGF-b1 | MR Egger | -0.159 | 0.119 | 0.853 | 0.708 | 1.028 |
| SLE | TGF-b1 | Weighted median | -0.013 | 0.839 | 0.987 | 0.871 | 1.119 |
| SLE | TGF-b1 | Inverse variance weighted | -0.032 | 0.475 | 0.968 | 0.886 | 1.058 |
| SLE | TGF-b1 | Simple mode | -0.115 | 0.317 | 0.891 | 0.718 | 1.108 |
| SLE | TGF-b1 | Weighted mode | 0.011 | 0.891 | 1.011 | 0.863 | 1.186 |
| SLE | IL-12 | MR Egger | -0.227 | 0.055 | 0.797 | 0.645 | 0.984 |
| SLE | IL-12 | Weighted median | -0.126 | 0.059 | 0.882 | 0.774 | 1.005 |
| SLE | IL-12 | Inverse variance weighted | -0.081 | 0.127 | 0.922 | 0.83 | 1.024 |
| SLE | IL-12 | Simple mode | 0.048 | 0.732 | 1.049 | 0.801 | 1.375 |
| SLE | IL-12 | Weighted mode | -0.187 | 0.076 | 0.83 | 0.686 | 1.004 |
| SLE | IL-10 | MR Egger | -0.525 | 0.322 | 0.592 | 0.335 | 1.046 |
| SLE | IL-10 | Weighted median | -0.088 | 0.555 | 0.915 | 0.683 | 1.228 |
| SLE | IL-10 | Inverse variance weighted | -0.005 | 0.977 | 0.995 | 0.729 | 1.36 |
| SLE | IL-10 | Simple mode | -0.136 | 0.583 | 0.873 | 0.58 | 1.315 |
| SLE | IL-10 | Weighted mode | -0.155 | 0.524 | 0.856 | 0.576 | 1.274 |
| SLE | IL-4 | MR Egger | -0.257 | 0.537 | 0.774 | 0.44 | 1.361 |
| SLE | IL-4 | Weighted median | -0.13 | 0.301 | 0.878 | 0.687 | 1.123 |
| SLE | IL-4 | Inverse variance weighted | -0.096 | 0.391 | 0.908 | 0.729 | 1.132 |
| SLE | IL-4 | Simple mode | -0.152 | 0.42 | 0.859 | 0.639 | 1.155 |
| SLE | IL-4 | Weighted mode | -0.15 | 0.426 | 0.861 | 0.64 | 1.158 |
| SLE | IFN-g | MR Egger | -0.005 | 0.96 | 0.995 | 0.827 | 1.197 |
| SLE | IFN-g | Weighted median | -0.093 | 0.121 | 0.911 | 0.809 | 1.025 |
| SLE | IFN-g | Inverse variance weighted | -0.027 | 0.547 | 0.973 | 0.892 | 1.062 |
| SLE | IFN-g | Simple mode | -0.099 | 0.358 | 0.905 | 0.738 | 1.111 |
| SLE | IFN-g | Weighted mode | -0.11 | 0.229 | 0.896 | 0.755 | 1.063 |
| SLE | IL-13 | MR Egger | -0.098 | 0.794 | 0.907 | 0.513 | 1.603 |
| SLE | IL-13 | Weighted median | -0.106 | 0.421 | 0.899 | 0.694 | 1.165 |
| SLE | IL-13 | Inverse variance weighted | -0.097 | 0.391 | 0.907 | 0.727 | 1.133 |
| SLE | IL-13 | Simple mode | -0.107 | 0.574 | 0.899 | 0.656 | 1.231 |
| SLE | IL-13 | Weighted mode | -0.107 | 0.549 | 0.899 | 0.671 | 1.204 |
| SLE | TGF-b3 | MR Egger | -0.187 | 0.069 | 0.829 | 0.69 | 0.998 |
| SLE | TGF-b3 | Weighted median | -0.079 | 0.207 | 0.924 | 0.817 | 1.045 |
| SLE | TGF-b3 | Inverse variance weighted | -0.051 | 0.279 | 0.95 | 0.866 | 1.042 |
| SLE | TGF-b3 | Simple mode | -0.044 | 0.689 | 0.957 | 0.775 | 1.182 |
| SLE | TGF-b3 | Weighted mode | -0.093 | 0.33 | 0.911 | 0.761 | 1.092 |
| SLE | TNF-a | MR Egger | 0.01 | 0.934 | 1.01 | 0.797 | 1.28 |
| SLE | TNF-a | Weighted median | 0.054 | 0.419 | 1.055 | 0.926 | 1.202 |
| SLE | TNF-a | Inverse variance weighted | 0.016 | 0.769 | 1.016 | 0.912 | 1.132 |
| SLE | TNF-a | Simple mode | 0.055 | 0.618 | 1.057 | 0.854 | 1.308 |
| SLE | TNF-a | Weighted mode | 0.084 | 0.381 | 1.088 | 0.906 | 1.306 |
| SLE | TGF-b2 | MR Egger | -0.04 | 0.708 | 0.961 | 0.785 | 1.177 |
| SLE | TGF-b2 | Weighted median | -0.01 | 0.87 | 0.99 | 0.875 | 1.119 |
| SLE | TGF-b2 | Inverse variance weighted | -0.038 | 0.422 | 0.963 | 0.878 | 1.056 |
| SLE | TGF-b2 | Simple mode | 0.032 | 0.747 | 1.032 | 0.855 | 1.246 |
| SLE | TGF-b2 | Weighted mode | -0.022 | 0.779 | 0.979 | 0.844 | 1.135 |
| SLE | IL-6 | MR Egger | 0.03 | 0.769 | 1.031 | 0.847 | 1.254 |
| SLE | IL-6 | Weighted median | 0.053 | 0.399 | 1.055 | 0.932 | 1.194 |
| SLE | IL-6 | Inverse variance weighted | 0.022 | 0.635 | 1.022 | 0.935 | 1.117 |
| SLE | IL-6 | Simple mode | -0.031 | 0.772 | 0.97 | 0.791 | 1.189 |
| SLE | IL-6 | Weighted mode | -0.041 | 0.683 | 0.959 | 0.79 | 1.166 |
| SLE | IL-1a | MR Egger | -0.015 | 0.887 | 0.985 | 0.799 | 1.213 |
| SLE | IL-1a | Weighted median | -0.075 | 0.246 | 0.928 | 0.817 | 1.053 |
| SLE | IL-1a | Inverse variance weighted | -0.032 | 0.503 | 0.968 | 0.88 | 1.065 |
| SLE | IL-1a | Simple mode | -0.117 | 0.364 | 0.89 | 0.697 | 1.136 |
| SLE | IL-1a | Weighted mode | -0.112 | 0.342 | 0.894 | 0.715 | 1.118 |

The results of MR were shown by approach of MR-Egger, Weighted median, Inverse variance weighted, Simple mode and Weighted mode. AS: ankylosing spondylitis; JME: juvenile myoclonic epilepsy; SLE: systemic lupus erythematosus; CI: confidence intervals; OR: odds ratio; MR, Mendelian randomization.

Supplementary Table S7 Mendelian randomization analysis of inflammatory cytokines and juvenile myoclonic epilepsy

| Exposures | Outcome | Method | Beta | P-value | OR | 95% CI-low | 95% CI-  up |
| --- | --- | --- | --- | --- | --- | --- | --- |
| TGF-b1 | JME | MR Egger | -0.0548 | 0.596 | 0.947 | 0.818 | 1.095 |
| TGF-b1 | JME | Weighted median | -0.007 | 0.283 | 0.993 | 0.98 | 1.006 |
| TGF-b1 | JME | Inverse variance weighted | -0.0053 | 0.323 | 0.995 | 0.984 | 1.005 |
| TGF-b1 | JME | Simple mode | -0.0079 | 0.374 | 0.992 | 0.979 | 1.006 |
| TGF-b1 | JME | Weighted mode | -0.0079 | 0.407 | 0.992 | 0.978 | 1.007 |
| IL-12 | JME | Inverse variance weighted | 0.0028 | 0.659 | 1.003 | 0.99 | 1.016 |
| IL-10 | JME | Inverse variance weighted | -0.0083 | 0.359 | 0.992 | 0.974 | 1.009 |
| IL-4 | JME | Inverse variance weighted | 0.0018 | 0.784 | 1.002 | 0.989 | 1.015 |
| IFN-g | JME | Wald ratio | 0.0044 | 0.559 | 1.004 | 0.99 | 1.02 |
| IL-13 | JME | Wald ratio | 0.0075 | 0.469 | 1.008 | 0.987 | 1.028 |
| TNF-a | JME | MR Egger | 0.0087 | 0.803 | 1.009 | 0.956 | 1.064 |
| TNF-a | JME | Weighted median | 0.0041 | 0.541 | 1.004 | 0.991 | 1.017 |
| TNF-a | JME | Inverse variance weighted | 0.0044 | 0.418 | 1.004 | 0.994 | 1.015 |
| TNF-a | JME | Simple mode | 0.0034 | 0.72 | 1.003 | 0.987 | 1.02 |
| TNF-a | JME | Weighted mode | 0.0035 | 0.697 | 1.004 | 0.988 | 1.019 |
| IL-6 | JME | Wald ratio | 0.0055 | 0.55 | 1.006 | 0.988 | 1.024 |
| IL-1a | JME | MR Egger | 0.0029 | 0.951 | 1.003 | 0.925 | 1.087 |
| IL-1a | JME | Weighted median | -0.0007 | 0.901 | 0.999 | 0.989 | 1.01 |
| IL-1a | JME | Inverse variance weighted | 0.0006 | 0.923 | 1.001 | 0.989 | 1.012 |
| IL-1a | JME | Simple mode | -0.0021 | 0.817 | 0.998 | 0.982 | 1.014 |
| IL-1a | JME | Weighted mode | -0.0018 | 0.818 | 0.998 | 0.985 | 1.012 |

The results of MR were shown by approach of MR-Egger, Weighted median, Inverse variance weighted, Simple mode and Weighted mode. JME: juvenile myoclonic epilepsy; CI: confidence intervals; OR: odds ratio; MR, Mendelian randomization.

Supplementary Table S8 Reverse Mendelian randomization analysis

| Exposures | Outcome | Method | Beta | P-value | OR | 95% CI-low | 95% CI-  up |
| --- | --- | --- | --- | --- | --- | --- | --- |
| JME | AS | MR Egger | 5.643 | 0.116 | 282.355 | 0.349 | 228707.003 |
| JME | AS | Weighted median | 0.671 | 0.593 | 1.956 | 0.167 | 22.885 |
| JME | AS | Inverse variance weighted | 0.592 | 0.516 | 1.807 | 0.302 | 10.797 |
| JME | AS | Simple mode | 3.632 | 0.183 | 37.797 | 0.22 | 6501.18 |
| JME | AS | Weighted mode | 3.685 | 0.185 | 39.831 | 0.208 | 7625.018 |
| JME | Crohn's disease | MR Egger | -0.065 | 0.975 | 0.937 | 0.017 | 50.671 |
| JME | Crohn's disease | Weighted median | -2.254 | 0.006 | 0.105 | 0.021 | 0.525 |
| JME | Crohn's disease | Inverse variance weighted | -1.034 | 0.104 | 0.356 | 0.102 | 1.236 |
| JME | Crohn's disease | Simple mode | -2.742 | 0.114 | 0.065 | 0.003 | 1.658 |
| JME | Crohn's disease | Weighted mode | -2.775 | 0.069 | 0.062 | 0.004 | 1.043 |
| JME | RA | MR Egger | 0.955 | 0.776 | 2.599 | 0.004 | 1708.273 |
| JME | RA | Weighted median | 0.831 | 0.217 | 2.296 | 0.613 | 8.596 |
| JME | RA | Inverse variance weighted | 1.799 | 0.031 | 6.047 | 1.175 | 31.128 |
| JME | RA | Simple mode | -0.132 | 0.92 | 0.876 | 0.068 | 11.347 |
| JME | RA | Weighted mode | -0.356 | 0.761 | 0.701 | 0.073 | 6.692 |
| JME | Sarcoidosis | MR Egger | 4.281 | 0.216 | 72.339 | 0.103 | 50845.65 |
| JME | Sarcoidosis | Weighted median | -0.234 | 0.834 | 0.791 | 0.089 | 7.028 |
| JME | Sarcoidosis | Inverse variance weighted | -0.169 | 0.848 | 0.844 | 0.149 | 4.783 |
| JME | Sarcoidosis | Simple mode | -0.02 | 0.993 | 0.98 | 0.013 | 71.475 |
| JME | Sarcoidosis | Weighted mode | -0.294 | 0.886 | 0.745 | 0.014 | 39.55 |
| JME | SLE | MR Egger | -2.997 | 0.217 | 0.05 | 0.001 | 4.601 |
| JME | SLE | Weighted median | 1.113 | 0.332 | 3.044 | 0.321 | 28.883 |
| JME | SLE | Inverse variance weighted | 0.465 | 0.567 | 1.592 | 0.324 | 7.816 |
| JME | SLE | Simple mode | 1.16 | 0.584 | 3.189 | 0.055 | 184.667 |
| JME | SLE | Weighted mode | 1.731 | 0.344 | 5.647 | 0.176 | 180.821 |
| JME | Type 1 diabetes mellitus | MR Egger | -0.649 | 0.921 | 0.523 | 1.65E-06 | 165285.987 |
| JME | Type 1 diabetes mellitus | Weighted median | 0.351 | 0.641 | 1.421 | 0.325 | 6.207 |
| JME | Type 1 diabetes mellitus | Inverse variance weighted | 1.7 | 0.383 | 5.476 | 0.12 | 250.307 |
| JME | Type 1 diabetes mellitus | Simple mode | -0.063 | 0.964 | 0.939 | 0.063 | 13.906 |
| JME | Type 1 diabetes mellitus | Weighted mode | -0.206 | 0.879 | 0.814 | 0.059 | 11.162 |
| JME | Ulcerative colitis | MR Egger | -1.897 | 0.257 | 0.15 | 0.006 | 3.603 |
| JME | Ulcerative colitis | Weighted median | -0.534 | 0.426 | 0.586 | 0.157 | 2.185 |
| JME | Ulcerative colitis | Inverse variance weighted | -0.317 | 0.531 | 0.728 | 0.27 | 1.966 |
| JME | Ulcerative colitis | Simple mode | 0.244 | 0.841 | 1.276 | 0.122 | 13.321 |
| JME | Ulcerative colitis | Weighted mode | 0.215 | 0.837 | 1.239 | 0.165 | 9.322 |

The results of MR were shown by approach of MR-Egger, Weighted median, Inverse variance weighted, Simple mode and Weighted mode. AS: ankylosing spondylitis; JME: juvenile myoclonic epilepsy; RA: Rheumatoid arthritis; SLE: systemic lupus erythematosus; CI: confidence intervals; OR: odds ratio; MR, Mendelian randomization

Supplementary Table S9 Sensitivity analysis of univariable and reverse Mendelian randomization

Heterogeneity test

| Exposure | Outcome | Heterogeneity test (IVW) | |
| --- | --- | --- | --- |
|  |  | (IVW)Q estimate | (IVW) Q P value |
| SLE | JME | 14.988 | 0.97 |
| Type 1 diabetes mellitus | JME | 29.407 | 0.167 |
| Sarcoidosis | JME | 5.426 | 0.143 |
| AS | JME | 1.373 | 0.927 |
| Celiac disease | JME | 16.065 | 0.025 |
| Crohn's disease | JME | 55.531 | 0.114 |
| Ulcerative colitis | JME | 29.772 | 0.192 |
| JME | SLE | 12.283 | 0.584 |
| JME | Type 1 diabetes mellitus | 243.84 | 1.98E-40 |
| JME | Sarcoidosis | 26.455 | 0.151 |
| JME | AS | 19.443 | 0.429 |
| JME | Crohn's disease | 25.618 | 0.141 |
| JME | Ulcerative colitis | 16.889 | 0.597 |

Horizontal pleiotropy test

| Exposure | Outcome | Horizontal pleiotropy test | | |
| --- | --- | --- | --- | --- |
|  |  | Intercept | P value | Globle test P value |
| SLE | JME | -0.0013 | 0.192 | 0.976 |
| Type 1 diabetes mellitus | JME | 0.0008 | 0.557 | 0.232 |
| Sarcoidosis | JME | -0.0134 | 0.192 | 0.177 |
| AS | JME | 0.0007 | 0.69 | 0.126 |
| Celiac disease | JME | 0.0025 | 0.128 | 0.134 |
| Crohn's disease | JME | 0.0002 | 0.831 | 0.225 |
| Ulcerative colitis | JME | 0.0005 | 0.735 | 0.935 |
| JME | SLE | 0.0439 | 0.133 | 0.55 |
| JME | Type 1 diabetes mellitus | 0.0288 | 0.707 | <0.001 |
| JME | Sarcoidosis | -0.0526 | 0.184 | 0.141 |
| JME | AS | -0.060 | 0.142 | 0.404 |
| JME | Crohn's disease | -0.0121 | 0.622 | 0.142 |
| JME | Ulcerative colitis | 0.0195 | 0.319 | 0.61 |

Sensitivity analysis of univariable and reverse Mendelian randomization

, including pleiotropy analysis and heterogeneity analysis. AS: ankylosing spondylitis; JME: juvenile myoclonic epilepsy; SLE: systemic lupus erythematosus.
